# Supplementary material for: Unveiling endothelial cell border heterogeneity: VE-cadherin adherens junction stratification by deep convolutional neural networks
Source: PLoS One. 2025 Jan 6;20(1):e0317110. doi: 10.1371/journal.pone.0317110 (PMC11703098; doi:10.1371/journal.pone.0317110)
Supplement: Attachment — Submitted filename: Second Rebuttal Coverletter.docx [file pone.0317110.s002.docx]

Dear Editor, Dear Reviewers,

We thank you kindly for the instructive feedback.

We have updated the manuscript to address the final concern of reviewer 2. Below we explain point by point how we addressed the concerns in the manuscript.

***Related to comparison (a), the figure referred to in the text when making these claims (Fig. 4) does not display quantitative data from individual replicates or a summary measure of the variance between replicates. (In contrast to comparison (b), in which each plasma sample is represented by a point in the PCA plot). Displaying replicates and/or and indicator of variance between replicates (e.g., error bars) is necessary. The authors also state in the methods “The differences between population of the clusters for different conditions were compared using Chi-square test, using a multiple technical replicates combined for the analysis.” Did authors truly combine (lump together) data from all replicates? These replicates define the variability of a given treatment and should be considered in the statistical analysis.***

We have altered our analysis to be able to use information from the technical replicates. We have therefore switched from using Chi-square test on the combined replicates (where we assumed combining technical replicates results in a better estimation of the true cluster belongings) to using the multivariate generalization of the T-test; the Hotelling’s t-squared test, combined with LDA analysis/visualization. Figure 4 is updated to include bars representing the standard deviating between replicates for each condition and cluster, and LDA analysis is shown to visualize how the replicates cluster together in the dimension reduced space. In addition, figure 5 has been updated to keep statistical approach the same between the two sections.

We highly appreciate your time and are looking forward to your response.

Sincerely,

Rudmer Postma

*On behalf of all co-authors*
